# Advanced glycation end products (AGEs) in relation to atherosclerotic lipid profiles in middle-aged and elderly diabetic patients

**DOI:** 10.1186/1476-511X-10-228

**Published:** 2011-12-06

**Authors:** Jin-Biou Chang, Nain-Feng Chu, Jhu-Ting Syu, An-Tsz Hsieh, Yi-Ren Hung

**Affiliations:** 1 Department of Medical Laboratory Science and Biotechnology, Yuanpei University, Hsinchu City, Taiwan; 2 Division of Clinical Pathology, Tri-Service General Hospital, National Defense Medical Center, Taipei, Taiwan; 3 Department of Community Medicine, Shuang-Ho Hospital, Taipei Medical University, New Taipei City, Taiwan; 4 School of Public Health, National Defense Medical Center, Taipei, Taiwan; 5 Department of Internal Medicine, Shuang-Ho Hospital, Taipei Medical University, New Taipei City, Taiwan; 6 Department of Internal Medicine, Division of Endocrinology, Tri-Service General Hospital, National Defense Medical Center, Taipei, Taiwan

## Abstract

**Objectives:**

To evaluate the association between AGEs and atherosclerotic lipid profiles among aging diabetic patients in Taiwan.

**Design and Methods:**

After age and gender matching, we selected 207 diabetic subjects and 174 diabetic subjects with proteinuria. Lipid profiles, including total cholesterol (TC), triglycerides (TG), high density cholesterol-lipoprotein (HDL-C) and low density lipoprotein-cholesterol (LDL-C) were measured using standard methods. AGEs were measured with the immunoassay method.

**Results:**

In general, males were heavier; however, females had higher AGEs, fasting glucose (GLU), TC, HDL-C and LDL-C levels than males, and had higher TC/HDL-C, LDL-C/HDL-C, and TG/HDL-C ratios compared to males. AGEs were more strongly correlated with TG levels and TCL/LDL-C, LDL-C/HDL-C and TG/HDL-C ratios when compared to glucose or hemoglobin A1c. Subjects had higher AGEs levels (≧ 2.0 AU) with more adverse lipid profiles.

**Conclusion:**

AGEs seem to be a good biomarker to evaluate the association between diabetes and atherosclerotic disorders in aging diabetes.

## Introduction

In the Framingham study, the mortality rate from cardiovascular disease (CVD) was two times higher among diabetic patients than the general population [1]. Similarly, in the Multiple Risk Factor Intervention Trial (MR-FIT), after an average of 12 years of follow up, the risk of mortality from cardiovascular disease was much higher among diabetic men than non-diabetic men, especially with higher CVD risk factor levels [2]. Furthermore, in the Munich General Practitioner Project, the major risk factors for macrovascular death among diabetic patients during ten years of follow up were age and glycosylated hemoglobin A1c levels [3].

Diabetes plays an important role in the development and occurrence of cardiovascular disease. Previous studies have clearly demonstrated that diabetes mellitus is one of the most important risk factors for atherosclerotic disorders; however, so far there is no good biological marker that can predict atherosclerotic lesions among diabetic patients. Diabetes may be associated with accelerated atherosclerosis by either increasing the conventional risk factors, such as dyslipidemia and high blood pressure, or diabetes-specific risk factors, such as advanced glycation end products (AGEs), reactive oxygen species (ROS) and matrix protein production [4]. Furthermore, the formation of AGEs may play a key role in diabetes and CVD, leading to chronic hyperglycemia, dyslipidemia and oxidative stress [5].

AGEs are the product of the initial glycation chain reaction in glucose metabolism [6,7]. In diabetes, hyperglycemia may be results in the cells unable to reduce glucose intake, which then results in mitochondrial production of reactive oxygen species and damaging of DNA. This may lead to inactivation and accumulation of the metabolites earlier in the metabolism pathway which includes over-production of AGEs. AGEs are reactive derivations of the Maillard process, from non-enzymatic glucose-protein condensation reactions between the glucose ketone groups and the amino groups of the protein, which may be associated with diabetes-related complications [8].

The purpose of this study is to evaluate the association between AGEs and atherosclerotic profiles among aging diabetic patients in Taiwan.

## Materials and methods

### Study subjects

All participating subjects were between 35 and 80 years old, ethnically Han Chinese, with type 2 diabetes mellitus, and were recruited from the outpatient clinic of the Tri-Service General Hospital in Taipei, Taiwan from July 2007 to Dec. 2009. After age and gender matching, we included 207 Type 2 diabetic patients without nephropathies (DM) and 174 diabetic patients with nephropathies (DN). All recruited patients fulfilled the following criteria: (i) age between 35 to 80 years old; (ii) fasting plasma glucose greater than 126 mg/dl; and (iii) HbA1c greater than 6%. The study subjects were further classified as DM or DN based on three surrogate endpoints: urinary albumin to creatinine ratio (ACR), BUN and serum creatinine. ACRs were measured more than twice at different time points. Patients who fulfilled any one of the following three criteria were classified in the DN group: (i) average ACR greater than 30 μg/mg; (ii) serum creatinine greater than 1.7 mg/dl; (iii) BUN greater than 20 mg/dl. The rest of the patients were classified as the DM group. According to this definition, micro- and macroalbuminuric patients were classified into the DN group. Approval was obtained from the Internal Review Board of Tri-Service General Hospital before conducting the study, and an approved informed consent form was signed by each subject.

### Biochemical variable measurement

Atherosclerotic profiles, such as total cholesterol (TC), triglycerides (TG), high-density lipoprotein cholesterol (HDL-C) and low-density lipoprotein cholesterol (LDL-C) levels were measured using standard methods. We also calculated the TC/HDL-C, LDL-C/HDL-C and TG/HDL-C ratios to represent the significance of the atherosclerosis. We further calculated the atherosclerotic index (AI) based on the distribution of TC, TG, HDL-C and LDL-C levels. For each subjects, we summed the points from TC (TC ≤ 200 = 1 point, 200 < TC ≤ 240 = 2 points, TC > 240 = 3 points), TG, (TG ≤ 150 = 1 point, 150 < TG ≤ 200 = 2 points, TG > 200 = 3 points), HDL-C (HDL-C ≥ 50 = 1 point, 50 > HDL-C ≥ 40 = 2 points, HDL-C < 40 = 3 points) and LDL-C (LDL-C ≤ 130 = 1 point, 130 < LDL-C ≤ 160 = 2 points, LDL-C > 160 = 3 points); the higher AI may be represented the more adverse atherosclerotic characteristics.

AGEs were measured using enzyme-linked immunoassay method (Hanson Hong Biomedical Co, Ltd, Taipei, Taiwan), using a Hitachi 7150 (Osaka, Japan) analyzer, and the within and between CV was 1.3% and 2.8%, respectively.

### Statistical analyses

All data were expressed as mean ± SD and the t-test was conducted to evaluate the differences between two groups. Spearman correlation coefficients were examined for fasting glucose (GLU), glycated hemoglobin A1c (A1c) and AGEs in relation to these atherosclerotic profiles and parameters. Multi-variable regression analyses were conducted to examine the association between AGEs and atherosclerotic profiles after adjusting for potential confounders, such as age and BMI, and further adjusting for GLU and A1c. We further evaluated the atherosclerotic characteristics of subjects with AGEs greater than or equal to, or less than 2.0 AU.

All statistical analyses were conducted using the statistical package SAS 8.2 (SAS institute Inc, Cary, NC, USA).

## Results

The general characteristics of study subjects by gender are shown in Table 1. In general, females had higher AGEs, A1C, TC, HDL-C and LDL-C levels than males (p < 0.05). However, males had higher TC/HDL-C, LDL-C/HDL-C, and TG/HDL-C ratios compared to females (all p < 0.05).

**Table 1:** General characteristics of study subjects.

	DM(N = 207)						DN(N = 174)					
	
	Male(N = 81)			Female(N = 126)			Male(N = 71)			Female(N = 103)		
	
	Mean	±	S.D	Mean	±	S.D	Mean	±	S.D	Mean	±	S.D
Age(yr)	63.5	±	8.7	62.4	±	7.2^y^	66.3	±	8.9	64.9	±	6.8
Weight(kg)	69.9	±	9.3***	61.6	±	10.0	70.0	±	9.3***	62.5	±	9.7
BMI(kg/m^2^)	25.4	±	2.9	25.3	±	3.7	25.3	±	3.2	26.2	±	3.9
AGEs(AU)	1.7	±	1.3	1.9	±	1.3^y^	1.9	±	1.5*	2.4	±	1.5
Glucose(mg/dL)	143.0	±	38.5	148.8	±	42.2	152.1	±	54.6	157.2	±	57.4
HbAlc(%)	7.9	±	1.4*	8.3	±	1.4^a^	8.4	±	1.7	8.8	±	2.0
CHOL(mg/dL)	177.4	±	32.7***	195.3	±	38.3	175.5	±	44.4***	203.0	±	39.4
TG(mg/dL)	140.6	±	75.5^a^	144.4	±	63.1^y^	179.7	±	125.0	189.0	±	106.8
HDL-C(mg/dL)	45.9	±	10.1***	56.1	±	13.2	43.2	±	10.2***	53.0	±	13.0
LDL-C(mg/dL)	114.4	±	30.4*	124.6	±	34.7	116.1	±	35.1*	129.8	±	38.2
CHOL/HDL-C	4.0	±	1.1*	3.7	±	1.1^y^	4.3	±	1.4	4.1	±	1.2
LDL-C/HDL-C	2.6	±	0.8	2.4	±	0.9	2.8	±	1.0	2.6	±	0.9
TG/HDL-C	3.4	±	2.3^a^	2.9	±	1.8^z^	4.6	±	3.6	4.0	±	2.7
AI	6.2	±	1.7	6.2	±	1.9^y^	6.7	±	1.9	6.9	±	1.8

* p < 0.05, ** p < 0.01, *** p < 0.001 when compared by gender.

^a^p < 0.05^b^p < 0.01^c^p < 0.001 when compared with different statuses in males

^x^p < 0.05^y^p < 0.01^z^p < 0.001 when compared with different statuses in females

BMI: body mass index; AGEs: advanced glycation end products; HbA1c: hemoglobin A1c; CHOL: cholesterol; TG: triglyceride; HDL-C: high-density lipoprotein-cholesterol; LDL-C: low density lipoprotein-cholesterol; AI: atherosclerotic index

Table 2 presents the Spearman correlation coefficients of fasting glucose, A1c and AGEs levels on lipid profiles and atherosclerotic characteristics among study subjects according to gender and disease status. When compared to GLU, A1c and AGEs, AGEs levels were more positively correlated with TG and TC/LDL-C, LDL-C/HDL-C and TG/HDL-C ratios (especially in males) and negatively correlated with HDL-C levels (all p < 0.01). These characteristics were present in both DM and DN subjects.

**Table 2:** Spearman correlation coefficient of fasting glucose, hemoglobin A1c (HbA1c) and advanced glycation end products (AGEs) with lipid profiles.

	DM(N = 207)						DN(N = 174)					
	
	Males(N = 81)			Females(N = 126)			Males(N = 71)			Females(N = 103)		
	
	Glucose	HbA1c	AGEs	Glucose	HbA1c	AGEs	Glucose	HbA1c	AGEs	Glucose	HbA1c	AGEs
Age(yrs)	-0.13	-0.15	-0.13	-0.12	-0.10	0.07	-0.40***	-0.10	-0.18	-0.01	0.03	-0.14
Weight(kg)	0.04	0.12	0.33**	0.23**	0.14	0.03	0.13	-0.08	0.27*	-0.07	0.11	0.12
BMI(kg/m^2^)	0.11	0.18	0.23*	0.18*	0.13	0.08	0.15	-0.09	0.29*	-0.08	0.05	0.11
CHOL(mg/dL)	0.10	0.21	0.12	0.34***	0.34***	0.34***	0.10	0.12	0.35**	0.26**	0.17	0.15
TG(mg/dL)	0.08	0.10	0.77***	0.12	0.24**	0.69***	0.25*	0.32**	0.73***	0.25*	0.26**	0.68***
HDL-C(mg/dL)	-0.02	-0.13	-0.34**	-0.07	0.03	-0.27**	-0.13	-0.17	-0.35**	0.08	0.04	-0.36***
LDL-C(mg/dL)	0.12	0.18	0.12	0.34***	0.35***	0.27**	0.11	0.13	0.19	0.19	0.15	0.03
CHOL/HDL-C	0.10	0.20	0.44***	0.30**	0.21*	0.51***	0.15	0.13	0.48***	0.10	0.09	0.41***
LDL-C/HDL-C	0.05	0.16	0.30*	0.32***	0.27**	0.40***	0.11	0.15	0.28*	0.13	0.14	0.26*
TG/HDL-C	0.11	0.11	0.73***	0.12	0.18	0.65***	0.26*	0.29*	0.72***	0.09	0.14	0.63***
AI	0.18	0.15	0.56***	0.29***	0.33***	0.51***	0.20	0.27*	0.63***	0.24*	0.22*	0.46***

*p < 0.05 **p < 0.01 ***p < 0.001

BMI: body mass index; AGEs: advanced glycation end products; HbA1c: hemoglobin A1c; CHOL: cholesterol; TG: triglyceride; HDL-C: high-density lipoprotein-cholesterol; LDL-C: low density lipoprotein-cholesterol; AI: atherosclerotic index

The multi-variable regression analyses of AGEs on lipid profiles and atherosclerotic characteristics, after adjusting for potential confounders, are presented in Table 3. In Model III, after adjusting for age, BMI, GLU and A1c levels, AGEs were positively associated with TC, TG, TC/HDL-C, LDL-C/HDL-C, TG/HDL-C and atherosclerotic index, and negatively associated with HDL-C levels in both genders.

**Table 3:** Multivariable regression models for the association between AGEs levels and atherosclerotic lipid profiles with gender-specificaiton.

		Model I	Model II	Model III
		
		β(S.E)	β(S.E)	β(S.E)
Males				
	CHOL(mg/dL)	7.9(2.5)**	7.7(2.5)**	7.4(2.6)**
	TG(mg/dL)	48.4(4.7)***	48.6(4.8)***	47.2(4.7)***
	HDL-C(mg/dL)	-2.7(0.6)***	-2.4(0.6)***	-2.3(0.6)***
	LDL-C(mg/dL)	2.7(2.1)	2.3(2.1)	2.0(2.1)
	CHOL/HDL-C	0.4(0.1)***	0.4(0.1)***	0.4(0.1)***
	LDL-C/HDL-C	0.2(0.1)**	0.2(0.1)**	0.2(0.1)**
	TG/HDL-C	1.4(0.1)***	1.4(0.1)***	1.3(0.1)***
	AI	0.8(0.1)***	0.8(0.1)***	0.8(0.1)***
Females				
	CHOL(mg/dL)	8.3(1.9)***	8.4(2.0)***	7.6(1.9)***
	TG(mg/dL)	41.8(3.4)***	40.9(3.4)***	40.0(3.4)***
	HDL-C(mg/dL)	-3.1(0.7)***	-3.0(0.7)***	-3.0(0.7)***
	LDL-C(mg/dL)	4.9(1.8)**	5.1(1.9)**	4.3(1.8)*
	CHOL/HDL-C	0.4(0.1)***	0.4(0.1)***	0.4(0.1)***
	LDL-C/HDL-C	0.2(< 0.1)***	0.2(< 0.1)***	0.2(< 0.1)***
	TG/HDL-C	1.1(0.1)***	1.0(0.1)***	1.0(0.1)***
	AI	0.7(0.1)***	0.7(0.1)***	0.7(0.1)***

Model I: after adjusting for age(yrs)

Model II: after further adjusting for age(yrs) and BMI

Model III: after further adjusting for age(yrs), BMI, fasting glucose and HbA1c

*p < 0.05 **p < 0.01 ***p < 0.001

BMI: body mass index; AGEs: advanced glycation end products; HbA1c: hemoglobin A1c; CHOL: cholesterol; TG: triglyceride; HDL-C: high-density lipoprotein-cholesterol; LDL-C: low density lipoprotein-cholesterol; AI: atherosclerotic index

We further divided subjects into two subgroups based on their AGEs levels (≧ 2.0 or < 2.0 AU). Table 4 shows the lipid profiles and atherosclerotic characteristics for different AGEs groups by gender. Subjects with AGEs ≧ 2.0 AU had higher TC, TG, LDL-C levels (in females), TC/HDL-C, LDL-C/HDL-C, TG/HDL-C ratios and atherosclerotic indexes in both sexes.

**Table 4:** General characteristics of study subjects by different AGEs levels.

	AGE ≦ 2.0 (N = 193)						AGE > 2.0 (N = 188)					
	
	Males(N = 88)			Females(N = 105)			Males(N = 64)			Females(N = 124)		
	
	Mean	±	S.D	Mean	±	S.D	Mean	±	S.D	Mean	±	S.D
Age(yrs)	65.6	±	8.9	63.3	±	7.4	63.8	±	8.8	63.4	±	6.9
Weight(kg)	67.9	±	9.3***^b^	60.5	±	9.8^x^	72.8	±	8.5***	63.3	±	9.8
BMI(kg/m^2^)	24.8	±	2.9^b^	25.2	±	3.8^x^	26.3	±	3.0	26.3	±	3.8
Glucose(mg/dL)	147.0	±	46.4	148.5	±	47.5	147.5	±	47.7	156.0	±	51.3
HbAlc(%)	8.1	±	1.6	8.3	±	1.6^x^	8.1	±	1.5*	8.8	±	1.8
CHOL(mg/dL)	170.4	±	36.5***^a^	189.2	±	32.9^z^	185.3	±	40.0**	206.9	±	41.8
TG(mg/dL)	110.0	±	64.0^c^	119.7	±	56.8^z^	226.0	±	109.4	202.2	±	92.5
HDL-C(mg/dL)	47.5	±	10.5***^c^	58.8	±	12.9^z^	41.1	±	8.6***	51.5	±	12.7
LDL-C(mg/dL)	113.6	±	29.5	120.4	±	30.6^x^	117.3	±	36.4*	132.4	±	39.7
CHOL/HDL-C	3.7	±	1.2*^c^	3.3	±	0.8^z^	4.7	±	1.1*	4.3	±	1.2
LDL-C/HDL-C	2.5	±	1.0*^b^	2.2	±	0.7^z^	3.0	±	0.9	2.7	±	1.0
TG/HDL-C	2.6	±	1.8^c^	2.2	±	1.2^z^	5.7	±	3.4**	4.4	±	2.5
AI	5.8	±	1.4^c^	5.8	±	1.5^z^	7.8	±	1.7	7.4	±	2.0
AI distribution	n (%)			n (%)			n (%)			n (%)		
0-4 (n = 119)	46(46.5)***			55(43.7)***			3(5.7)			15(14.6)		
5-8 (n = 190)	46(46.5)			59(46.8)			30(56.6)			55(53.4)		
9-12 (n = 72)	7(7.1)			12(9.5)			20(37.7)			33(32.0)		

* p < 0.05, ** p < 0.01, *** p < 0.001 when compared by gender.

^a^p < 0.05^b^p < 0.01^c^p < 0.001when compared with different statuses in males

^x^p < 0.05^y^p < 0.01^z^p < 0.001 when compared with different statuses in females

BMI: body mass index; AGEs: advanced glycation end products; HbA1c: hemoglobin A1c; CHOL: cholesterol; TG: triglyceride; HDL-C: high-density lipoprotein-cholesterol; LDL-C: low density lipoprotein-cholesterol; AI: atherosclerotic index

## Discussion

In this study we examined different glucose metabolism variables, such as fasting glucose (GLU), glycated hemoglobin A1c (A1c) and AGEs. We found AGEs levels correlated positively with lipid profiles and atherosclerotic characteristics, and this correlation was even better than those for fasting glucose or A1c in both sexes. Even after adjusting for age, BMI, GLU and/or A1c and other potential confounders, AGEs are still significantly correlated with lipid profiles and atherosclerotic characteristics. More interestingly, the subjects with higher AGEs levels had worse lipid profiles, more adverse atherosclerotic characteristics and higher AI. AGEs thus seem to be a good biomarker for evaluating the association between diabetes and atherosclerotic disorders among aging diabetic patients.

There are several interesting findings in this study, but also some limitations. First, diet and physical activity were not well controlled in this study. It may be associated with non-differential misclassification which could attenuate the results, but we still found significant findings. Second, the duration and control of diabetes was not considered and adjusted for in the final analyses, but we could estimate that the higher AGEs or A1c, which may relate to poor control of diabetes and then processed more significant atherosclerotic lesions. However, the lipid profiles and atherosclerotic indexes were indirect biomarkers for atherosclerosis; the gold standard methods of diagnosis, such as coronary angiography angiography, should be considered to evaluate coronary artery lesions in the future. Third, the cross-sectional study design may limit our exploration. Long-term follow-up study can provide more specific causal relationships between AGEs and atherosclerotic diseases. Finally, inflammation may be a potential confounder for the relation between AGEs and atherosclerotic status; but we did not measure inflammatory markers, such as high sensitivity C-reactive protein, interleukin-6 and tumor necrosis factors in this study. Further comprehensive study of biomarkers is indicated to identify the potential mechanisms.

Many studies have shown that cardiovascular diseases with rapid progression of atherosclerosis are the most important causes of death in diabetes mellitus [4,9-11]. Other studies have shown that patients with diabetes have high incidence of atherosclerotic diseases [2]. The accelerated atherosclerosis in diabetic patients may be explained by the hypothesis that human LDL-C oxidation plays a role in the initiation of atherosclerosis [11-13]. Duration and/or control of diabetes may relate to later DM-induced complications, but it remains controversial whether these include micro- or macro-vascular lesions [1,9]. However, most previous studies suggested that A1c is one of the best indicators to evaluate diabetes control and predict the development of diabetic-related complications [14]. In this study, we found that AGEs were associated positively with total cholesterol, TG, LDL-C and other atherosclerotic indexes, and correlated negatively with HDL-C levels, which suggests that AGEs may be a marker for atherosclerosis in diabetic patients. Furthermore, in this study, these associations were even stronger than fasting glucose or A1c levels. These findings indicate that not only AGEs-modified forms of LDL-C but also AGEs-peptides contribute to tissue injury and the development of atherosclerosis in diabetic patients [11,12].

These findings were similar to other studies, which indicate that AGEs might be associated with either diabetes control or diabetes-related complications. In addition, AGEs have been reported to be linked with the effects of previous glycemic control and the subsequent developments of micro- and macro-vascular complications in diabetes [6]. Furthermore, serum AGEs levels are increased when patients have type 2 diabetes and coronary artery disease (CAD) [15]. The association between AGEs and diabetes-related complications may not only due to hyperglycemia per se but also the products of AGEs. A study by Kanauchi and his colleagues showed that AGEs may participate in the development of CAD, even in non-diabetic participants [16]. However, other study showed that AGEs may significantly impair LDL-receptor-mediated clearance which contributes to the elevation of LDL-C levels in patients with diabetes or renal insufficiency [12]. Furthermore, AGEs may also contribute to the development of cardiovascular diseases through the modification of the lipid profiles [12]..

AGEs are present in coronary atheroma of both diabetic and non-diabetic patients [17,18]. Concentrations of circulating AGEs are correlated to the severity of coronary artery disease and the adverse clinical outcomes [19]. AGEs are linked to atherosclerosis in the following potential mechanisms. First, AGEs promote protein cross-linking, which leads to the covalent trapping of pro-atherogenic particles such as LDL in the arterial wall [11,20]. Second, excessive LDL trapping by hyperglycemia-induced AGEs may be associated with the accelerated development of atherosclerosis in patients with diabetes [11]. Third, AGEs may bind to apolipoprotein B particles which impaired their hepatic clearance, induced retention of LDL in the aortic wall and increased recognition by macrophages at this site, that all associated with the increased localization of AGEs-LDL in vessel wall and increased production of foam cells and accelerating atheroma formation [18,21]. Lastly, AGEs may induce oxidant stress by enhancing free radical generation from neutrophils. Furthermore, AGEs might enhance neutrophil NADPH oxidase activity and contribute to the vascular oxidant stress and cardiovascular disease in diabetic, uremic and elderly patients [22]. As indicated in this study, AGEs may be one of the better biomarkers to predict atherosclerosis in diabetic patients.

We found that there was a different association between AGEs and lipid profiles for males and females, not only in patients with DM alone, but also in DM patients with nephropathy. For example, after adjusting for potential confounders, the association between AGEs levels and atherosclerotic profiles was stronger in females than males. This result was similar to other studies that reported AGEs levels were significantly associated with mortality from all causes and coronary heart disease mortality in women, but not in men [19]. The associations between AGEs and lipid profiles were different for the two sexes which suggested that AGEs may play a role as a competing risk factor in atherosclerotic lesions when compared to other traditional CVD risk factors. In females, AGEs are a relatively important factor associated with lipid profiles, but in males, other factors--such as cigarette smoking, alcohol consumption and lower HDL-C--may be more closely associated with atherosclerotic lesions and lipid profiles. Otherwise, AGEs may represent an additional risk for CVD only in relatively low-risk subjects [19].

More interestingly, there was a closer association between AGEs and lipid profiles and atherosclerotic index in patients with DM nephropathy than those with DM alone. A possible explanation is that the patients with DM nephropathy may have more serious illness and higher AGEs levels, which may make them more prone to develop DM-related atherosclerosis. However, further studies are necessary to examine the pathophysiology and to understand the possible mechanisms.

Our results show the association between AGEs and lipid profiles persisted even after adjusting for potential confounders, and further adjusting for fasting glucose or A1c levels, which suggested that there might evidence to demonstrate that AGEs are potentially a better predictor of atherosclerotic lesions in diabetic patients than fasting glucose or A1c. These findings were similar to other studies asserting that AGEs may be associated with either control of diabetes or complications of diabetes, and also linked them with diabetes-related complications such as atherosclerosis and restenosis after percutaneous coronary intervention [23-25]

There is an age-dependent increase of serum AGEs levels, especially after age 13 [26,27]. In this study, we divided the patients into two subgroups based on their AGEs levels (≥ 2 and < 2 AU). The AGEs ≥ 2 patients were heavier, had larger BMIs and higher total cholesterol and TG levels than patients with AGEs < 2. These findings are similar to other studies showing that subjects with higher AGEs had more adverse atherosclerotic profiles than normal subjects [26].

We found that patients with higher AGEs had more adverse atherosclerotic parameters and indexes, which suggests that AGEs could be used as a marker to predict atherosclerotic lesions. However, further studies are necessary to evaluate the clinical relevance and clinical application of AGEs testing, especially for the prevention of diabetes-related atherosclerotic diseases.

## Competing interests

The authors declare that they have no competing interests.

## Authors' contributions

JBC performed the data collection, statistical analyses, and participated in drafted the manuscript. NFC carried out the design, coordination and conduction of the study, participated in the data collection and analysis, participated in drafted the manuscript. JTS participated in the conduction of the study, data collection, and statistical analysis. ATH performed the data collection, statistical analyses, and participated in drafted the manuscript. YRH carried out the design and coordination of the study. All authors read and approved the final manuscript.
